# Tracing pathways: the impact of the polycrisis on mental health trajectories in emerging adulthood

**DOI:** 10.3389/fpubh.2026.1782194

**Published:** 2026-07-07

**Authors:** Paulo Pinheiro, Yudit Namer, Anna Christina Nowak

**Affiliations:** 1 Centre for Prevention and Intervention in Childhood and Adolescence (CPI), Faculty of Educational Science, Bielefeld University, Bielefeld, Germany; 2 Department of Psychology, Health and Technology (PHT), Faculty of Behavioural, Management and Social Sciences (BMS), University of Twente, Enschede, Netherlands; 3 School of Public Health, Bielefeld University, Bielefeld, Germany; 4 Institute for Interdisciplinary Research on Conflict and Violence, ConflictA – Konfliktakademie, Bielefeld University, Bielefeld, Germany

**Keywords:** emerging adulthood, identity formation, polycrisis, social inequality, transactional framework, youth mental health

## Abstract

The accelerating convergence of global crises, including climate change, pandemics, geopolitical conflict, forced migration, digital transformation, and rising social inequality, has fundamentally reshaped the developmental environment for emerging adults (ages 18–29). Positioned at the critical juncture of identity formation and societal integration, this cohort faces unprecedented structural and psychological challenges. While scientific discourse, exemplified by the recent Lancet Psychiatry Commission, increasingly links macro-level megatrends to deteriorating mental health in this group, the specific transmission mechanisms remain analytically challenging. Building on this discourse, the article proposes a conceptual systematization of these pathways. The crisis concept is expanded beyond objective events to encompass crisis as a permanent narrative and a mechanism of ideological disruption. A critical reflection addresses the Western-centric bias in precarity discourse and conceptualizes the “polycrisis” as a syndemic that exacerbates vulnerabilities differently across the Global North and South. By integrating structural precarity, erosion of the symbolic order, and mechanisms underlying compromised transitions, a multi-layered transactional framework is outlined. This model links systemic disruptions to the dual developmental tasks of identity formation and social integration, providing an analytical lens to understand how structural incoherence contributes to individual psychopathology.

## 1 Introduction: a generation in transition

The transition to adulthood has historically marked a period of profound individual change. Conceptualized by Arnett (1) as “Emerging Adulthood,” this distinct life stage (spanning approximately ages 18–29) is culturally defined by five key features: identity exploration, instability, self-focus, feeling “in-between,” and a sense of infinite possibilities. Ideally, it is a Eriksonian moratorium characterized by “volitional exploration,” where young people navigate educational and professional choices with a high degree of agency (2). It involves a divergence between earlier sociocultural maturity and prolonged socioeconomic dependency (3), culminating in the critical tasks of consolidating a worldview and assuming roles within civil society (4).

However, the idea of an open horizon of possibilities, which underlies this developmental model, is currently being challenged. Today's emerging adults face a phase fundamentally reshaped by an unprecedented context of global instability. The current generation encounters not only material crises such as climate change, pandemics, armed conflicts, mass displacement, economic volatility, rising inequality, democratic backsliding und authoritarian consolidation, as well as digital transformation, but also a pervasive sense of crisis as a permanent condition. What makes this constellation distinctive is not merely the emergence of multiple stressors, but their interlinked mutually reinforcing character and their reach into domains that previously served as stabilizing background conditions. The current polycrisis combines threats to material living conditions with an informational crisis that can destabilize shared reality itself. In an environment shaped by platform dynamics and algorithmically curated content, uncertainty is not only about what will happen, but also about what is real, trustworthy, and collectively actionable. This instability is not merely cyclical but structural, indicating a global systemic shift toward precarity as a defining feature of future life (5). For emerging adults, this has developmental consequences that go beyond additional obstacles. The pathway to a good life may appear precarious and increasingly undefined, lacking shared meaning and credible scripts of progress. This can fracture meaning and agency by weakening future imagination, long term planning, and hope. While earlier generations also faced major challenges, the present context is distinctive in its simultaneity, global interdependence, and digitally accelerated amplification, producing cascading disruptions that reduce perceived controllability and limit intergenerational guidance. Consequently, the “volitional exploration” originally described by Arnett is increasingly constrained, resulting in “forced navigation” through precarious terrain. Inherently, previous generations can offer only limited guidance and act as role models, as they did not have to negotiate comparable combinations of disruptions within their own transition to adulthood.

Addressing the mental health of emerging adults has therefore become a critical global concern, especially given epidemiological evidence that around two-thirds of all mental disorders have their onset by age 25 (6). Prominent voices in the field, such as the Lancet Psychiatry Commission (7), have underscored the urgency of this issue, pointing to the detrimental role of “megatrends” and the polycrisis. While this recognition represents meaningful progress, it prompts further theoretical consideration of how macro-societal factors are transmitted to the micro-level of individual experience. Establishing this link is analytically demanding. It requires translating global abstractions like “ideological erosion” or “climate change” into the specific vocabulary of developmental psychology and psychopathology.

This article uses the current focus on macro-determinants as an occasion to propose a conceptual systematization of these transmission mechanisms. Addressing these pathways effectively calls for collaboration across disciplinary boundaries. The analysis suggests that the polycrisis constitutes a qualitatively distinct developmental environment that intersects with and accelerates longstanding social and health inequalities. It fundamentally shapes the social fabric in which the core developmental tasks of identity formation and social integration are enacted, thereby creating conditions of heightened vulnerability not through individual deficit, but through systemic incoherence.

## 2 The polycrisis revisited: crisis concepts, narratives, and the digital layer

Understanding the transmission from macro to micro levels requires a more nuanced view of systemic pressures. The terminology describing contemporary challenges has evolved alongside increasing recognition of their interconnectedness. The term “polycrisis” refers to the simultaneous occurrence of multiple crises, whose interactions generate synergistic harms that far exceed the sum of their individual effects (8). However, “crisis” itself is not an unambiguous term. According to the approach of reflexive crisis research (9), it is necessary to distinguish between crisis as an event and crisis as a communicative construction. In the lives of emerging adults, these dimensions intersect in several ways:

- Objectively experienced crises: immediate, tangible threats such as extreme weather events, viral outbreaks, forced displacement, or armed conflicts, which generate direct material harm.
- Crisis as a permanent state: the normalization of uncertainty, often described as “chronic crisis” or “permacrisis” (10, 11). In this context, crisis is no longer perceived as a singular event with a clear beginning and end, but rather becomes an enduring, omnipresent condition. This can be understood as a persistent background factor shaping everyday life.
- Crisis as narrative and spectacle: as Grimm et al. (9) illustrate, crisis is also a discursive construct employed by hegemonic institutional actors to mobilize resources or to structure collective meaning.
- Crisis as rupture and destabilization of shared reality: contemporary crises increasingly erode shared reality by weakening trust in how information is produced, validated, and circulated in digitally saturated media environments. This can foster chronic uncertainty and fragmentation that compromises coherent future oriented meaning making.

For emerging adults, these dimensions often co-occur but are processed in different ways. They do not simply accumulate; instead, they hold varying degrees of subjective relevance and may activate distinct psychological pathways. While objective crises require immediate adaptation, narrative crises primarily influence the interpretation of the future and can be internalized permanently. Both forms, however, are continually mediated through language and specific institutional actors. This includes political entities that employ crisis narratives to justify structural adjustments or demand individual resilience, as well as global media and tech corporations that benefit from the monetization of psychological arousal.

A defining aspect of this mediation is digitalization. Here, digital transformation is not regarded as an isolated trigger, but as the primary environment in which the polycrisis is experienced. Digital platforms accelerate the dissemination of crisis narratives, transforming global catastrophes into immediate, personal psychological stressors (12, 13). The algorithmic structure of social media amplifies processes of polarization and “doomscrolling.” This blurs the distinction between real-world risks and their narrative anticipation (14). This dynamic may intensify depending on how artificial intelligence systems are deployed, particularly when they are used to automate the production, targeting, and rapid distribution of misleading, fabricated, or context stripped content. In such environments, if reliable verification becomes harder to achieve, crisis may be not only anticipated but experienced as cognitively inescapable, weakening perceived agency and reducing the capacity to maintain stable and shared imaginaries of a desirable future. As a result, a “crisis of the imagination” can emerge, whereby the constant digital connectedness leads to a state in which “crisis” is experienced as a pervasive, continuous condition of alertness. This state is closely linked to chronic hyperarousal, hypervigilance, and an increased risk of anxiety disorders.

## 3 Global realities and the “precarity” trap: de-centering the perspective

A nuanced systematization should consistently acknowledge the Western-centric bias often present in youth research. The very concept of “emerging adulthood” is not a universal biological phenomenon, but rather a socio-historical product of the late 20th century (15). It emerged specifically in affluent societies, where the massive expansion of higher education since the 1970s and the shift toward knowledge economies allowed and required the postponement of traditional adult roles. This phase of “prolonged exploration” relies on a specific infrastructure of wealth and social security that is largely absent outside the context of Western, Educated, Industrialized, Rich, and Democratic (WEIRD) societies (16). Applying this framework universally risks obscuring local realities where early transition into work remains the norm.

Similarly, the concept of “precarity” requires critical reflection. In the Global North, the polycrisis is experienced as a biographical disruption, representing a frightening departure from postwar stability and a loss of entitlement and privilege. This dynamic fosters what can be termed “status anxiety” (17). In contrast, for many people in the Global South and for marginalized populations worldwide, precarity has historically been a systemic norm rather than a new exception (18). However, to avoid a purely deficit-based perspective, it is crucial to recognize that precarity in the Global South is not merely a narrative of deprivation. While Munck (18) underscores that structural precarity has long been a systemic condition in many Southern contexts, research on aspirations and hope in development settings suggests that chronic adversity can coexist with future-oriented agency that is socially and culturally mediated (19, 20). Compared with the “status anxiety” prevalent in parts of the Global North, collective practices of mutual support and political struggle can provide meaning-making resources and practical coping capacities without negating the structural violence involved. Acknowledging such agency therefore does not romanticize precarity. Rather, it sharpens the argument that the polycrisis acts as an inequality accelerant by shifting the heaviest burdens of adaptation onto those with the least institutional support, even where collective resources remain comparatively strong.

This is most visibly manifested in the experience of migration and displacement, where the polycrisis forces young people to navigate not only developmental transitions but also physical relocation, the loss of citizenship rights and often mistranslations of their life skills. Therefore, the polycrisis is understood as an “inequality accelerant” across both local and global scales.

- Locally: Within Western societies, socioeconomic status, sociocultural background, and gender remain principal axes of vulnerability, confirming established gradients of health equity (21). Privileged groups possess “crisis capital” (financial reserves and social networks) to buffer against shocks. Disadvantaged groups, including women and gender minorities (22), face “multiplied vulnerability,” where a single shock can precipitate a downward spiral. Empirical analysis validates this accelerating divergence: during the COVID-19 pandemic, for instance, the mental health gap between advantaged and disadvantaged young adults widened significantly (23). This vulnerability is most pronounced in forms of extreme precarity, such as experiencing homelessness, which is strongly correlated with severe psychopathology (24).
- Globally: The “polycrisis” reinforces long-standing structural asymmetries. The resources for adaptation are concentrated in the North, while the risks (climate impact, resource conflicts and wars) are exported to the South, driving forced migration (25).

For mental health, this implies that while the feeling of crisis may be global (mediated digitally), the capacity to cope is radically unequal. Recognizing this prevents the pathologization of resilience strategies used in the Global South and by refugee populations, which are often invisible within Western individualistic frameworks.

## 4 Developmental mechanisms: compromised integration and vulnerability

To fully grasp the impact of these global and local pressures, the specific mechanism of vulnerability inherent to this life stage must be differentiated from earlier developmental phases. While a “first window of vulnerability” in adolescence is primarily driven by internal neurobiological maturation (26), a “second window” in emerging adulthood is characterized by a psychosocial mismatch between developmental tasks and environmental instability (27, 28). This phase constitutes a decisive juncture for structural integration, relying on stable institutional pathways to translate individual capacity into social standing (29, 30). However, the polycrisis systematically erodes the material basis of these transitions. Vulnerability arises from the collision between the persistent normative demand for autonomy and an environment of structural exclusion (29).

In this context, the central developmental task is fundamentally altered. Whereas successful integration was once predicated on following a standard biography, emerging adults now face “compromised” transitions (31). Under stable conditions, exploration leads to commitment. However, within the context of the polycrisis, this process often devolves into “ruminative exploration” (32), a repetitive and anxious cycle of questioning without resolution. Clinically, this inability to plan long-term traps the individual in a state of stagnation, a condition strongly associated with depressive symptoms and generalized anxiety (33). It transforms the productive search for self into an incapacitating state of worry, making the necessity for constant, short-term adaptation not only a social requirement, but also a direct source of psychological pathology.

## 5 Tangible pathways: exemplary evidence of transmission

These theoretical mechanisms translate into tangible risks within the lives of emerging adults. Current empirical research substantiates this, providing exemplary evidence of how the polycrisis acts as a syndemic: a synergistic interaction of health risks and socio-structural adversity (34) that exacerbates pre-existing vulnerabilities. Three specific domains highlight these correlations.

### 5.1 Climate crisis and the global-local tension

The climate crisis exemplifies how global threats become individual psychological burdens, commonly referred to as eco-anxiety (35). Studies indicate that this response is not pathological but an adaptive reaction to a real existential threat, as younger generations are disproportionately exposed to climate extremes compared to previous cohorts (36). This phenomenon highlights the acute tension between global awareness and local constraints: young people recognize existential risks but are often limited by local structures that restrict their agency. Although eco-anxiety is often considered a “Western” issue, the material impacts are unevenly distributed. Climate change disproportionately affects communities in the Global South, where it represents not only future anxiety but an acute existential challenge. Nevertheless, the narrative of climate doom is shared globally, fostering a sense of “intergenerational betrayal” (37). Psychologically, this erodes fundamental trust in a safe future, a core component of mental stability, and paves the way for chronic stress and existential dread.

### 5.2 Pandemics and social fragmentation

The COVID-19 pandemic provides a clear empirical case of a syndemic crisis, where consequences were profoundly shaped by pre-existing social disparities (38). For emerging adults, it severed critical social ties necessary for identity development. Crucially, the impact was mediated by the digital divide. Access to reliable internet and hardware determined whether digitalization functioned as a buffer or an amplifier of exclusion (39). The pandemic highlighted that “digital sociality” is now a prerequisite for social integration. The loss of physical spaces for interaction led to a unique form of “social atrophy.” This disruption of social scaffolding is associated with increased rates of loneliness and depressive disorders, as the crucial peer-mirroring necessary for identity consolidation was absent (40). At the same time, the long-term significance of crises is demonstrated by COVID-19 even after they have subsided. The level of mental health has not yet returned to pre-pandemic levels (41, 42).

### 5.3 Geopolitical conflicts and the rise of the “navigational self”

Geopolitical instability and economic volatility intensify a sense of external locus of control (43). Research indicates that these pressures fundamentally disrupt identity formation by creating a normative dissonance. Societal institutions continue to propagate the ideal of a “planning self,” suggesting that adherence to linear biographical paths guarantees stability. However, the volatile reality forces emerging adults to enact a “navigational self” (10, 44), characterized by short-term tactical maneuvering rather than long-term strategic commitment. This mode of navigation aligns with the Western societal ideal of the “entrepreneurial self” (45), yet it comes at a high psychological cost. As critiques of crisis socialization suggest (46), this discourse shifts the logic of crisis management inward: structural failure to provide security is reinterpreted as an individual failure to be sufficiently resilient. This dynamic has a dual consequence:

- Internally, it places the burden of managing systemic risk onto the individual, fostering shame and self-blame (47), which significantly increases the risk for burnout and depressive episodes.
- Externally, the conflict between the entitlement of stability and the necessity of constant adaptation is experienced as a violation of the biographical contract. When societal structures demand long-term planning but fail to provide the predictability required for it, trust in these institutions erodes. Consequently, young adults may respond by turning away from established civic structures, viewing them as deceptive. Clinically, this “institutional mistrust” fosters profound cynicism and hopelessness, reducing the willingness to invest in a future that appears fundamentally unreliable.

## 6 Toward a systematization: a multi-layered transactional framework

The relationships described above point to the need for a more cohesive analytical model. Substantiating the link between megatrends and mental health outcomes, as discussed in recent research (7), goes beyond simple description and calls for a heuristic systematization to trace the transmission of macro-societal shifts into the microstructure of individual development. To address this, an integrated, multi-layered transactional framework is outlined. This model does not view the individual in isolation but situates the developing subject within a nested system of ideological, digital, and social forces.

### 6.1 Layer 1: the symbolic order—the fracture of meaning and agency

A central tenet of this framework is the epistemological position that psychological distress in the polycrisis cannot be understood solely as a reaction to material threats. Instead, it must be analyzed as a symptom of a fractured symbolic order (48). Defined simply, the symbolic order is the “invisible infrastructure of meaning” that holds a society together. It encompasses the unwritten rules, shared narratives, language, and laws that make social reality appear consistent, predictable, and “normal.” It acts as a container for internal representations of past, present and future selves. It functions as a collective map, translating complex events into a coherent story of how the world works and where one fits into it.

Currently, however, emerging adults are witnessing the disintegration of this map. The dominant cultural script continues to demand performance and optimism, while material reality increasingly signals decline and ecological decay. Berlant (49) describes this dynamic as “cruel optimism.” This rupture creates a “symbolic dissonance”: the map no longer matches the territory. This fracture offers a compelling analytical lens to approach the paradox of “informed inertia”: Why do emerging adults now, despite being the most crisis-aware generation, often exhibit political paralysis or a retreat into conservative and right-wing patterns? Mark Fisher's concept of “capitalist realism” (50) provides a valuable heuristic here. It invites us to consider whether this inertia is not a failure of will, but a structural foreclosure of imagination. Fisher argues that the pervasive ideology creates a sense that “there is no alternative,” effectively canceling the future as a space of radical difference.

Viewed from this perspective, the observed paralysis might be understood as a rational response to an environment where the symbolic order precludes the imagination of a different world and of a future self capable of shaping it. If the future is framed merely as a worsening of the present, agency is reduced to individual survival. This perspective helps explain why distress often manifests not as progressive action, but as depressive stagnation or a defensive retreat. Here, the link to salutogenesis serves as the clinical connector, identifying the symbolic order as the prerequisite for the sense of coherence (51). Consequently, the incoherence of the symbolic map precipitates the erosion of the pillars of health: meaningfulness is challenged by a future that appears narratively “foreclosed,” manageability dissolves as systemic risks prove insoluble through individual resilience, and comprehensibility fractures as the promise of endless growth contradicts the material reality of limits. Clinically, this manifests as a profound “crisis of agency.” The subject suffers from the inability to translate knowledge into transformative action, resulting in a sense of entrapment.

### 6.2 Layer 2: social and digital ecologies—the empirical interface

The crisis of the symbolic order does not impact the individual directly but is mediated through specific social environments. Drawing on and expanding ecological systems theory (52), this layer serves a dual function: theoretically, it differentiates how systemic pressures are filtered; methodologically, it acts as the primary interface for empirical operationalization. While the abstract dissonance of layer 1 requires qualitative critique, the ecological systems at layer 2 offer quantifiable variables that allow for measuring the specific impact and pathways of the polycrisis reaching the individual.

- Macrosystem (ideological and structural): This layer encompasses the overarching institutional patterns and ideologies. Currently, a specific tension is observed: the ideological demand for the “entrepreneurial self” (autonomy, agility, self-optimization) persists (45), while the structural supports (social security systems, distinct career ladders) are eroding.
- Exosystem (the socio-digital ecology): This layer acts as the immediate context of exposure, characterized by a dual dynamic of material erosion and digital saturation:

- Material erosion: physically, the infrastructure for transitioning to adulthood is shrinking. Exploding housing costs and the casualization of local labor markets (gig economy) create a physical environment of instability, often referred to as “generation rent” (53). Furthermore, the loss of accessible “third places” (54), such as community centers and public spaces, removes the physical scaffolding for social cohesion.
- Digital saturation: as physical spaces contract, the digital sphere expands to fill the void. Through the “attention economy” (55), crisis narratives are monetized and algorithmically amplified. This creates a feedback loop: the digital sphere fragments the shared reality necessary for social cohesion while simultaneously serving as the primary (and often toxic) coping space for the loss of physical community.

- Microsystem (immediate experience): This level comprises the immediate environments in which emerging adults live and interact daily (family, peer groups, intimate partnerships, workplace). Here, the polycrisis is not discussed as an abstract concept but is enacted as interpersonal reality. Systemic instability infiltrates these spaces, manifesting as relational stress or social friction. This transmission can take diverse forms, ranging from economic precarity straining romantic relationships to political polarization fracturing friendship networks, or the general “spillover” of systemic anxiety into daily social interactions.

### 6.3 Layer 3: psychological mediation—the translation of experience

This layer functions as the crucial psychological translation mechanism. It explains how the external deficits measured in layer 2 are internalized within the subjective experience of the self, a process in which socialization acts as the primary conduit for health inequalities (56). While layer 2 describes the environment, layer 3 describes the lived reality of this environment through the lens of basic human needs and the everyday interpretive work through which people make their lives coherent under constraint.

Individuals are active agents who attempt to navigate these environmental contradictions (57). Yet, when the prospect of social integration is undermined, this negotiation process becomes significantly impaired. In a polycrisis context, marked by interacting economic, political, ecological, and social disruptions, this translation from structural conditions to subjective experience is unlikely to be linear. Macro level instability does not simply add stress. It can reshape the conditions under which planning, commitment, and identity continuity remain viable.

#### 6.3.1 SDT as a need based micro vocabulary, useful but not exhaustive

The internalization of such experiences can be elucidated using Self-Determination Theory (SDT) (58), which highlights how societal conditions influence the satisfaction of basic psychological needs. Within this framework, the polycrisis systematically frustrates the fulfillment of these needs (autonomy, competence, and relatedness) which are essential for successful integration and psychological wellbeing. However, the scale and convergence dynamics of polycrisis arguably require an additional conceptual move. The current environment may not merely frustrate these needs but fundamentally reconfigure the possibility of agency itself, that is, the degree to which self directed action can be sustained, socially recognized, and converted into stable life trajectories.

#### 6.3.2 Beyond SDT: socio structural coherence and the biographical contract

To address this interplay between macro and micro processes more directly, this paper therefore proposes a complementary framework of “socio structural coherence.” In this view, mental health is not only a matter of internal need satisfaction, but also the outcome of a functioning biographical contract between the individual and society. This contract refers to a minimally reliable alignment between effort and reward, between participation and recognition, and between present actions and plausible future returns. In other words, people can tolerate strain when the social world remains sufficiently intelligible and when credible pathways exist through which agency can translate into acknowledged roles.

The polycrisis can be conceptualized as a systemic breach of this biographical contract. When the symbolic order as outlined in layer 1 fractures, the individual is not just less autonomous. They may be forced into a condition of intensified responsibilization, in which they are implicitly required to internalize global structural failures as personal responsibility and manage them as private psychological burdens. This suggests a shift from need frustration, in SDT terms, to a more foundational disturbance in existential security, namely ontological insecurity. Following Giddens (59), ontological security refers to a basic sense of continuity, trust, and reliability in the self and the social world. Ontological insecurity, by contrast, denotes a persistent weakening of this felt continuity and trust, including the erosion of taken for granted expectations that make everyday planning, social belonging, and identity stability possible. Under polycrisis, the self can become less a site of growth and more a site of constant crisis management. In that stance, the ongoing task is not development but containment, not aspiration but damage control. Crucially, this breach is not merely cognitive but social and recognitional. When individuals cannot access socially legible roles or have their contributions recognized as valid, agency no longer converts into standing, belonging, and self worth. Misrecognition and contested membership thus operate as mechanisms through which structural exclusion becomes psychologically consequential (60).

#### 6.3.3 Proximate SDT mechanisms linking polycrisis to distress

Within SDT's proximate mechanism, the risk of psychological distress increases when:

- Autonomy is undermined by precarity (5), which often dictates life choices rather than allowing for intrinsic self-direction. This loss of agency is a primary precursor to learned helplessness and depressive affect. Under conditions of socio structural incoherence, the problem is not only diminished choice but weakened confidence that choices matter, meaning that effort is reliably connected to outcomes in a recognizable social trajectory.
- Competence is challenged not only by skill deficits, but also by structural exclusion. When young adults, including those with refugee backgrounds, cannot find meaningful roles in the labor market or civil society, or when their skillsets are not recognized in the new setting, they experience “role deprivation” and invisibility (61). This directly undermines self-worth, increasing vulnerability to depressive symptoms. In socio structural coherence terms, this is precisely where the biographical contract fails. Competence does not translate into socially validated placement, eroding both efficacy and future orientation.
- Relatedness is fractured by social polarization and digital fragmentation. The resulting social isolation is a robust risk factor for suicidal ideation and anxiety disorders. Layer 1 dynamics, particularly symbolic fracture, matter here because they can turn everyday differences in values, lifestyles, or group identities into morally charged distinctions between “us” and “them.” Once differences are framed in moral terms, they become boundaries that regulate who is seen as legitimate, trustworthy, or deserving of inclusion. This destabilizes feelings of belonging and weakens mutual recognition as a taken for granted social norm.

#### 6.3.4 Integrative conclusion: from compromised integration to ontological insecurity

This “compromised integration” forces a corrosive reevaluation of self-concept. The inability to inhabit recognized social roles translates socio structural exclusion into internal feelings of worthlessness and pathology. The added value of the socio structural coherence perspective is that it clarifies why polycrisis may yield not only more distress but potentially a qualitative shift in vulnerability patterns. When the biographical contract is repeatedly breached, distress is organized not merely around frustrated needs, but around destabilized continuity, recognition, and credible futures.

### 6.4 Layer 4: intrapsychic outcomes—responses and pathologies

This final layer represents the endpoint where structural deficits manifest in the individual. Here, the framework differentiates between psychosocial responses, understood as active behavioral attempts to manage the crisis, and clinical pathways, which mark the transition into psychopathological decompensation.

#### 6.4.1 Psychosocial responses: the spectrum of coping

Faced with “symbolic dissonance” (layer 1) and “compromised integration” (layer 3), emerging adults adopt specific strategies to regain agency. These responses form a spectrum ranging from constructive engagement to defensive withdrawal.

- Transformative agency (restoring meaning): Where “crisis capital” is available, emerging adults may respond with collective action (e.g., climate activism). Psychologically, this counters the “cancellation of the future” by actively rewriting the narrative. Collective efficacy acts as a buffer against helplessness, restoring a sense of meaningfulness and social connection despite structural instability (62, 63).
- The navigational self (functional adaptation): As described in 4.3, this refers to the attempt to remain functional through hyper-adaptability. The individual focuses on short-term tactical maneuvering (“surfing the crisis”). While functional, this mode often comes at the cost of long-term identity consolidation and leads to chronic exhaustion (10).
- Distress (affective resonance): For many, a primary response is acute emotional distress such as “eco-distress” or “solastalgia” (64). This response reflects a high degree of permeability to the crisis; the individual remains emotionally open to the reality of loss. While painful, this distress is not inherently pathological but can be viewed as a rational emotional response to objective threat. However, without channels for agency, it risks devolving into paralysis (65).
- Indifference (defensive resignation): Where the breach of the biographical contract is perceived as irreparable, emerging adults may respond with defensive hardening. This “social coldness” (*bürgerliche Kälte*) is a strategy to minimize pain by withdrawing emotional investment from societal institutions (“silent quitting”) (66).
- Radicalization (regressive agency): When the loss of comprehensibility becomes unbearable, individuals may seek artificial certainty. According to Uncertainty-Identity Theory, radical ideologies serve as a “prosthetic symbolic order,” offering rigid rules and exclusionary group identities to compensate for the lost sense of coherence (67).

#### 6.4.2 Clinical pathways: the crystallization of distress

When coping strategies are insufficient, structural strain may contribute to the emergence of specific psychopathological trajectories. However, these processes are neither linear nor uniform; instead, they are shaped by dynamic interactions between individual vulnerabilities and contextual factors. Longitudinal data indicate that for emerging adults, mental health deterioration often does not peak during the acute crisis phase, but rather manifests subsequently, suggesting a process of cumulative “crisis fatigue” (68). Building on Layers 1–3, this section specifies how the previously described mechanisms can consolidate into clinically salient patterns over time. In clinical terms, symbolic fracture can translate into a persistent loss of ontological security, meaning a weakening of the experienced continuity and reliability of self and social world. Likewise, repeated breaches of the biographical contract can consolidate into learned helplessness and depressive cognition, particularly when effort no longer appears predictably linked to stability or recognized roles. In such contexts, volatility undermines the capacity to sustain a planning oriented self, and systemic failure is more readily internalized as personal inadequacy.

Potential trajectories include:

- The anxiety trajectory: Characterized by chronic uncertainty and digital hyperarousal, individuals may remain in a persistent state of hypervigilance. Clinically, this can manifest as Generalized Anxiety Disorder or panic symptoms, reflecting a somatic attempt to manage an uncontrollable environment (13).
- The depressive trajectory: When the “navigational self” collapses under the pressure of self-optimization or when agency is persistently obstructed, depressive disorders may develop. In these cases, internalized structural failure can lead to shame and a pathology of agency, such as learned helplessness (47).
- The loneliness trajectory: Processes of atomization and the loss of communal spaces frustrate the fundamental need for relatedness. This may result in clinical loneliness, a profound form of isolation distinct from solitude and a robust predictor of suicidal ideation and behavior (69).

These trajectories are not mutually exclusive and may co-occur or shift over time, reflecting the complex and dynamic nature of psychological adaptation under conditions of prolonged structural strain.

## 7 Discussion and outlook: bridging the macro-micro gap

Current psychiatric discourse, as exemplified by the recent Lancet Psychiatry Commission (7), has recognized the polycrisis as a critical determinant of youth mental health. Nevertheless, a substantial theoretical gap persists: while the “what” (megatrends) is increasingly acknowledged, the “how” (the mechanisms through which these trends are internalized) remains underexplored. Prevailing models tend to conceptualize societal crises predominantly as external stressors, analogous to natural disasters, which risks neglecting their specific character as crises of meaning, coherence, and institutional reliability, as well as their digitally mediated and chronic nature. This perspective article seeks to address this gap by positing that a robust understanding of emerging adult psychopathology necessitates a perspective systematically attuned to societal macrodynamics and their psychological mediation. The proposed framework endeavors to integrate sociological ideology critique with developmental psychology, suggesting that contemporary mental health pathologies may be interpreted less as individual dysfunctions and more as intelligible responses to structural incoherence and compromised processes of integration. While the framework offers considerable heuristic potential, its practical implementation requires careful methodological reflection. A central challenge lies in operationalizing abstract constructs such as “symbolic dissonance” or “ideological foreclosure” within empirical research, particularly in quantitative paradigms. Bridging the gap between abstract sociological theory and clinical methodology calls for instrument innovation and interdisciplinary study designs that extend beyond prevailing research conventions. At the same time, the polycrisis may resist the degree of reductionism typically required for measurement. It is not a single exposure but a dynamic configuration where the most salient exposure is likely driven by chronic anticipatory uncertainty, cumulative institutional unreliability, and digitally amplified crisis narratives. Explicitly acknowledging this tension is essential to avoid overly simplistic proxies that collapse meaning-related mechanisms into generic stress indicators.

In light of these considerations, progress is unlikely to emerge from a single comprehensive study. Instead, the framework invites cumulative and modular testing of specific links between layers, ideally through converging evidence across methods and contexts. This implies methodological pluralism, including qualitative work on meaning-making and lived experience, quantitative longitudinal designs capable of modeling accumulation and temporal ordering, and intensive approaches that capture the socio-digital interface. Across designs, particular care is needed to distinguish context-appropriate distress from clinically significant impairment and to avoid pathologizing rational responses to structural threat.

Three flexible entry points appear especially promising: (7) mapping socio-digital ecologies as proximal contexts of exposure (Layers 2 and 3), (1) decentering and comparative research beyond WEIRD settings to specify how the “biographical contract” is locally negotiated (Layers 3 and 4), and (2) tracing phenomenological and clinical pathways through which macro-level meaning disruptions and institutional mistrust consolidate into syndemic vulnerabilities over time (Layers 1 and 4). Ultimately, sustained collaborative and interdisciplinary work is required. Within the scope of this Research Topic, the aim is to assemble a broad range of conceptual, methodological, and empirical contributions that help map the scope of emerging-adult distress in the context of the polycrisis. Rather than delivering definitive tests of the framework, the collected articles are intended to surface convergences and productive tensions across disciplines, sharpen key constructs, and make operationalization challenges visible. Considered collectively, these contributions can inform a more targeted set of hypotheses, measures, and study designs for subsequent research and thereby support the iterative refinement of the proposed framework.

## Data Availability

The original contributions presented in the study are included in the article/supplementary material, further inquiries can be directed to the corresponding author.
